# Youth Resilience to Drought: Learning from a Group of South African Adolescents

**DOI:** 10.3390/ijerph17217896

**Published:** 2020-10-28

**Authors:** Linda Theron, Motlalepule Ruth Mampane, Liesel Ebersöhn, Angie Hart

**Affiliations:** 1 Department of Educational Psychology, Centre for the Study of Resilience, University of Pretoria, Pretoria 0027, South Africa; Ruth.mampane@up.ac.za (M.R.M.); Liesel.ebersohn@up.ac.za (L.E.); 2 Centre of Resilience for Social Justice, School of Health Sciences, University of Brighton, Brighton BN2 4AT, UK; a.hart@brighton.ac.uk

**Keywords:** African adolescents, climate change, co-productive approach, protective community, social ecological theory of resilience

## Abstract

Exposure to drought is on the increase, also in sub-Saharan Africa. Even so, little attention has been paid to what supports youth resilience to the stressors associated with drought. In response, this article reports a secondary analysis of qualitative data generated in a phenomenological study with 25 South African adolescents (average age 15.6; majority Sepedi-speaking) from a drought-impacted and structurally disadvantaged community. The thematic findings show the importance of personal, relational, and structural resources that fit with youths’ sociocultural context. Essentially, proactive collaboration between adolescents and their social ecologies is necessary to co-advance socially just responses to the challenges associated with drought.

## 1. Introduction

Worldwide, young people’s health and wellbeing is threatened by continuing climate change and related phenomena, such as drought [1]. Drought, which has been termed ‘a slow-moving disaster’, p. 13252 [2], is on the rise [3]. It is associated with heightened youth risk for impaired hygiene, poor health, psychological distress, and disrupted schooling [4,5,6,7]. Moreover, drought typically exacerbates co-occurring stressors, such as poverty or social inequity [8]. Although multiple studies have detailed the drought-related health and wellbeing risks [2], few have investigated the factors that protect youth health and wellbeing in the face or aftermath of drought. Put differently, few have investigated what enables youth resilience to drought-related risks. Given predictions that exposure to drought is unlikely to subside any time soon [1,3] and concomitant admonitions that ‘building resilience to drought remains a critical concern’ [9], the inattention to drought-related youth resilience is problematic.

Such inattention is particularly concerning in South Africa. Like much of sub-Saharan Africa, South Africa is drought prone [10]. The recurrence of drought makes it essential to support South African youth to adjust well to this problematic reality. Like much of sub-Saharan Africa, South Africa’s youth population is also substantial: one third of South Africa’s population is adolescent; almost half its population is younger than 25 [11]. This population’s health and wellbeing is, however, challenged by multiple risks, including climate change phenomena, widespread unemployment and associated disadvantage, and high incidence of crime and violence. The resilience of this sizeable population—also in the face of drought—matters for their capacity to contribute meaningfully to South Africa’s socioeconomic capital and future development [12].

In order to redress the inattention to what enables youth resilience to drought-related risks, this article reports the insights of 25 South African adolescents about the factors that they consider to be protective of youth health (physical and mental) in the face of drought. This purpose also redresses concerns that the resilience literature has tended to marginalise the voices, so to speak, of youth [13], more especially youth living in low- and middle-income countries in the Southern hemisphere [14]. The youth-directed insights that this article reports are likely to benefit all those interested in championing youth resilience to drought (i.e., supporting health and wellbeing outcomes among youth exposed to drought), particularly those who work with African youth and are attentive to how responsive resilience processes are to sociocultural context.

### 1.1. Youth Resilience

The precise meaning of resilience continues to be debated. However, there is widespread endorsement of systemic understandings of youth resilience as a process that draws on personal and social-ecological resources to support normative development or healthy functioning in the face or aftermath of a significant stressor [15,16,17,18,19,20]. Personal resources include the capacity to regulate emotion and behaviour, be agentic, and value future-orientation [15]. Whilst meaningful connections to others are dominant social-ecological resources [21,22], there is growing recognition that structural and sociocultural resources also matter for resilience [23]. In addition to understanding youth resilience as a process that requires more than just personal resources, social-ecological accounts of resilience also acknowledge that the process is sensitive to contextual determinants [18]. For this reason, it is possible that the resources associated with the resilience of youth from one context will be less meaningful to the resilience of youth from a dissimilar context [24]. Similarly, because there is some understanding that gender shapes resilience processes [25], it is possible that resources associated with the resilience of girls will be less meaningful to boys and vice versa.

A systematic review of the 2009–2017 studies of the resilience of South African children and youth confirmed that South African young people’s resilience is grounded in personal and social-ecological (i.e., relational, structural and cultural) resources [20]. This review also drew attention to contextually specific resources, such as traditional African rites of passage and the valuing of human interdependence (e.g., norms and practices relating to Ubuntu). Subsequent South African studies have continued to demonstrate the salience of personal and social-ecological resources to youth resilience. For instance, Bireda and Pillay reported that personal resources (e.g., future aspirations), relational supports (e.g., supportive families, supportive peers), structural resources (e.g., a well-functioning school, spaces to play) and cultural capital (e.g., faith-based practices) were foundational to the resilience of 10 school-attending adolescents challenged by HIV-related stressors [26]. Similarly, personal resources (e.g., future aspirations, positive sense of self) and social-ecological ones (e.g., positive connections to peers and teachers, social services, and faith-based practises) were associated with the resilience of 23 IsiXhosa-speaking, adolescent orphans [27].

#### Youth Resilience to Drought

Youth—including sub-Saharan youth—are particularly vulnerable to drought-related risks and the impact of such risks on health and wellbeing [3]. For instance, a study [28] of 83,990 partnered women living in 19 sub-Saharan countries found strong associations between drought and intimate partner violence (IPV)—specifically physical and sexual violence—among adolescent girls (15 to 19-year-olds). Adolescent girls were also at higher risk than older women for reporting emotional violence during or after drought exposure. Whilst adolescent girls are generally considered to be especially vulnerable to IPV as a result of their youth and relational inexperience, the authors concluded that drought (and associated income and food insecurities) worsened these risks. Similarly, lower educational attainment and higher HIV rates were associated with adolescent girls and young women (15–24 years) living in drought-impacted rural areas in Lesotho [29]. Male youth are not immune to drought-related risks either. For instance, food insecurity, household tension, loss of educational opportunities, and related psychological distress were reported by the 30 adolescents (12 boys; 18 girls) who participated in a qualitative study on their experiences of drought in Botswana [30].

Despite how drought exposure challenges adolescent health and wellbeing, relatively few studies have investigated youth resilience to drought or included youth in studies that are broadly interested in resilience to drought. The exceptions [4,6,7,31] affirm that personal and social-ecological resources have resilience-enabling value in the face of drought too. Amongst others, the identified resources included young people having confidence in their own capacity to cope with drought-related challenges, a sense of humour, opportunities for employment, and social networks. Access to mental health information and counsellors that understood the challenges of drought exposure, youth-focused support groups, and a community that collaborated to beat drought-related challenges (e.g., shared fund-raising initiatives) were also key. Although these studies provide useful insight into the personal and social-ecological resources that matter for youth resilience in contexts of drought, their generalisability is limited by the fact that all youth participants were Australian. Given that resilience is a contextually sensitive process [15,18], it cannot be assumed that the Australian findings will be relevant to youth living in sub-Saharan Africa.

There is a dearth of studies investigating the resilience of youth living in drought-challenged sub-Saharan contexts. Whilst her study was not resilience focused per se, Babugura [30] did report some “coping mechanisms” (p. 144) that adolescents used to manage negative drought experiences in Botswana. These included acceptance of the inevitability of drought and prayer, as well as searching for work or providing sexual favours to cope with food- and income-insecurity associated with drought. There was also appreciation of government initiatives, including feeding schemes, that lessened the negative impact of drought on their households. Again, given that resilience is a contextually sensitive process and changeable over time [15,18], it is unclear how relevant Babugura’s findings will be a decade later to a sample of youth in a drought-challenged South African community.

## 2. Materials and Methods

To compensate for the inadequate attention to the factors that support youth resilience to drought, this article draws on data from the *Patterns of resilience among young people in a community affected by drought* study. The study followed a qualitative design. In particular, this article reports a secondary analysis of a subset of the qualitative data, namely the dataset generated in April 2017. We (the authors) were all involved in the generation and analysis of this data subset. The concerns associated with secondary analysis of qualitative data—such as an inadequate understanding of the study context, ethical data use, or insufficient rigour—are reduced when researchers involved in the primary study and analyses are also involved in the secondary analysis [32,33,34].

Secondary analyses of qualitative data are frequently concerned with supplementing or extending pre-existing analyses [33]. The pre-existing analyses were spearheaded by post-graduate students who collaborated in the *Patterns of resilience* study. They documented these analyses in their unpublished master’s dissertations [35,36,37,38,39]. These same analyses informed policy-directed outputs. In summary, these pre-existing analyses either provided narrowly focused accounts (e.g., accounts that were limited to the role of spirituality or family in youth resilience to drought) or broad descriptions that were not purposefully attentive to a range of social-ecological factors (i.e., relational, structural, and sociocultural) and their alignment with contextual dynamics. Accordingly, the secondary analysis was expressly concerned with personal and social-ecological factors that protected adolescent health and wellbeing during exposure to drought, and their fit with the study context.

### 2.1. Primary Study

#### 2.1.1. Context

The study was conducted in the municipality of Govan Mbeki, Mpumalanga Province. Like many other provinces in South Africa [10], Mpumalanga is drought prone [40]. Specifically, there are regular reports of inadequate rainfall for Govan Mbeki and significant water shortages [41,42,43]. Govan Mbeki [44] is home to 83,874 households, of which just over 12% are agricultural. In addition to drought-related challenges, Govan Mbeki reports low rates of education (only 31% of its adult residents have completed secondary school) and socioeconomic disadvantage (26% of its residents are unemployed; 24% of households own a computer; 36% own a car; 74% have a refrigerator). People who are challenged by socioeconomic disadvantage are especially vulnerable to the risks of drought and other climate change phenomena [45].

#### 2.1.2. Participants

As explained elsewhere [46], youth ‘participants’ took on roles as co-researchers in this study rather than identifying as participants. This fit with the study’s goal of university-affiliated researchers co-producing knowledge with community members. Although the university-affiliated researchers took on a directive role in the data generation that informs this article, the co-researchers took the lead in subsequent policy-directed activity and outputs.

A nonprofit organisation with which the lead author (L.T.) has a long-standing research collaboration facilitated co-researcher recruitment via their Govan Mbeki office. They pinpointed a community within the municipality as particularly vulnerable to drought, given its dependence on agriculture and long-term exposure to structural disadvantage. In addition to residence of this community at the time of the study, co-researcher eligibility was determined by age (i.e., 15–24 years old) and capacity to communicate in English.

Fifty co-researchers were recruited; 43—all African—accepted the invitation. This article draws on the insights of the 25 that were aged between 15 and 18 (nine adolescent boys, 16 adolescent girls; average age: 15.6). The choice to exclude the older co-researchers relates to the understanding that emerging adults (i.e., young people in the 18–29 year range) are developmentally different from adolescents [47]. All 25 were school attending at the time of the study. The majority reported that Sepedi was their home language.

#### 2.1.3. Ethics

The Ethics Committee, Faculty of Education, University of Pretoria granted ethical approval (UP 16/11/02). All adolescent co-researchers and their caregivers provided written consent. Co-researchers indicated whether their insights should be reported under their first name or anonymously (i.e., via a self-chosen pseudonym). Although the research team made provision for psychological support to co-researchers (i.e., it included a project manager with counselling experience and three Educational Psychologists), this was not utilised as none of the research activities elicited psychological distress. Co-researchers received a supermarket voucher of modest monetary value as a token of appreciation for their co-generation of knowledge.

#### 2.1.4. Data Generation

The research team used a variety of participatory visual methods to support co-researchers to share their lived experiences of drought and resilience to drought. As explained by de Lange [48], participatory visual methods are respectful of co-researchers’ expert understanding of the research phenomenon and position them as co-creators of knowledge. The methods use a specific prompt to invite creation of visual artefacts that give voice, as it were, to co-researchers’ knowledge. To this end, co-researchers were invited to use a range of materials (e.g., paper, crayons, pencils, clay, beads, sand, figurines) to make an artefact (e.g., a drawing, clay model, picture in the sand, body map) that responded to prompts that encouraged them to share their knowledge of drought and resilience. As is typical of the collaborative principles informing visual participatory work [49], co-researchers directed researcher understanding of what the artefacts meant by explaining the meaning of what they had produced and participating in related discussion.

For the purposes of the current article, the following prompts were pertinent: How does drought affect you? When there is a drought, what helps you stay healthy in your body, mind, and heart? What/who makes it possible for young people to be OK when there is drought? The references to being healthy or OK fit with the synonyms for resilience used in pre-existing South African studies of child and adolescent resilience [50].

To generate data, the adolescent co-researchers met university-affiliated researchers in the community hall and self-divided into groups of 4–6. As reported in previous studies of resilience, groupwork is a useful format when working with youth [14,51]. As the hall was spacious, no group was cramped. Data generation took approximately 4 h (excluding breaks for refreshments and lunch).

Each group was facilitated by a master’s student and co-facilitated by an honour’s student or youth member of the research team (in addition to academics from South Africa and Britain, the team included youth affiliated to Boingboing, a UK centre that uses co-productive methods to advance youth and community resilience). The authors trained the facilitators and co-facilitators to use visual participatory methods and facilitate related discussions, including how to probe for rich detail. The explanations and discussions of the meaning of the artefacts were recorded and transcribed by the facilitators. The transcriptions were checked for accuracy by the project manager.

#### 2.1.5. Data Analysis

The facilitators coded the transcripts inductively. To do so, they identified data relating to how drought affected the co-researchers and to factors and processes that supported co-researchers to manage these effects constructively. Following Creswell [52], they assigned open codes to the identified data (i.e., short labels that summarised the content of that data segment). The facilitators then met to compare their coding. Supported by the authors, they engaged in consensus discussions to resolve differences in coding [53]. They grouped similar codes and assigned relevant axial codes to each group (i.e., a label that represented the thematic coherence of the grouped codes; 52). Thereafter, they grouped the axial codes into themes and sub-themes (with illustrative excerpts from the transcripts) and included these in visual summaries that informed member checking sessions (June 2017) with the co-researchers. They also engaged co-researchers in member checking sessions. These offered broad confirmation of the primary analysis.

### 2.2. Secondary Analysis

The first author (L.T.) conducted a secondary analysis of the data. Following Stuckey [54], this entailed using deductive or a priori lenses to analyse the data. A social-ecological perspective of resilience (i.e., the understanding that resilience is a contextually sensitive process that draws on personal strengths and social-ecological resources; [15,23]) informed the deductive lens. More specifically, the four resilience-enablers reported in the systematic review by Van Breda and Theron [20], i.e., personal, relational, structural and spiritual/cultural resilience enablers and their associated categories, were used as deductive codes. Using these codes, L.T. searched for data that included reference to personal resilience-enablers (i.e., to agency, adaptive meaning-making, adaptive dispositional qualities, commitment to education, self-regulation, self-esteem, physical advantages); relational resilience-enablers (i.e., affective support, opportunities for growth/development, instrumental support); structural resilience-enablers (i.e., financial supports, community facilities/services, community safety, school systems); and spiritual/cultural resilience-enablers (religious/spiritual beliefs; enabling cultural values; enabling cultural practices). These data were labelled to explain how the identified resilience-enabler supported youth resilience to drought. Thereafter she grouped similar labels and assigned summative thematic labels. For instance, labels relating to adolescent actions to save water were summarised as water-wise agency. When it became apparent that the themes were mostly about health-promoting tactics and water-conserving ones, these two broad groupings were used as thematic clusters.

To heighten trustworthiness, L.T. searched for evidence that did not fit neatly with the identified themes (i.e., engaged in negative case analysis; [55]) and invited an experienced resilience researcher who was not affiliated to the study to audit the findings. Finally, the co-authors reviewed the findings. Neither the audit nor the co-author review resulted in substantive changes.

## 3. Results

**Figure 1:**
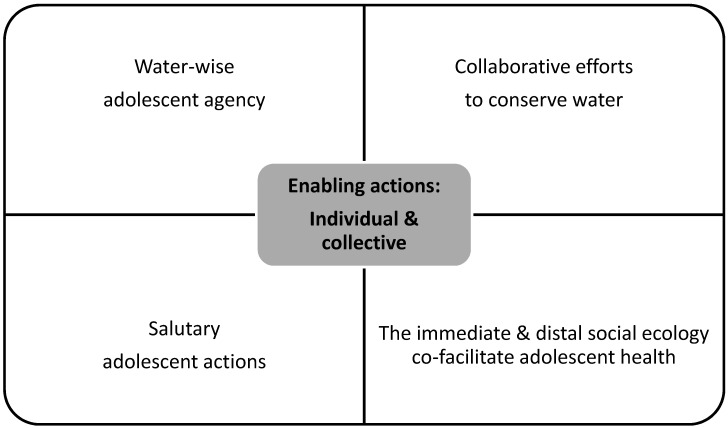
Summary of findings.

Enabling strategies—i.e., constructive efforts to manage the physical and psychosocial effects of drought—predominated co-researchers’ understandings of what supported resilience to drought-related hardship. These enabling strategies were actions that comprised health-promoting tactics and water-conserving ones. Both operated at the level of the adolescent and adolescent’s social ecology (see Figure 1). Evidence for the strategies was saturated, in that a majority of co-researchers (i.e., 80% plus) reported the identified strategies. The strategies were reported similarly, regardless of co-researchers’ age, sex, or level of schooling.

Before detailing the strategies reported in Figure 1, it is important to delineate participants’ experience of drought-related hardship. KidEazy (adolescent boy) summarised this hardship as: ‘*There was no water and we were suffering*’. As explained by others, such suffering had multiple dimensions including food-insecurity and escalating food prices: ‘*Most of our parents are like—they’re working as farmers and uh when … drought strikes we have the problem that uh … the crops are starting to die*’ (Thato, adolescent girl); ‘*We did all experience that food was starting to be expensive, and each and everything was starting to be expensive*’ (Kutlo, adolescent girl). In the absence of nutritious food and water supplies, physical health deteriorated: ‘*There’s no longer money to buy food … we can’t cook, you can’t wash your clothes, you can’t bath and you’re going to… get infections*’ (Khanyisile, adolescent girl) and ‘*People are coughing, they are dry and they are sick*’ (Angel, adolescent girl). Education was disrupted too: ‘*People can’t go to school … where are they going to get water … to cook, to wash their bodies before they go… they can’t go to school and work stinking*’ (Junior, adolescent boy); ‘*When I come back from school, I … go to this place [to fetch water], like it was far, and I must study at that time*’ (Hakeem, adolescent boy); and ‘*It was two weeks before we were about to write our exams, final exams … then at 10 o’clock we knock off [schools closed due to water shortage] … some of us failed the year exam coz …there was a syllabus that was not completed*’ (Nocebo, adolescent girl).

### 3.1. Water-Conserving Strategies

Water-conserving strategies were efforts—both reactive and proactive—to not waste what little water there was and/or to store water when this was possible. Whilst some of these efforts were driven by adolescents, others were collaborative and featured adolescents in partnership with their immediate social ecology. There was scant mention of distal social ecology efforts to support water conservation.

#### 3.1.1. Water-Wise Adolescent Agency

Co-researchers reported purposefully using less water when water was scarce. For example, Boka said: ‘*I learn a lot when there is drought … I learn to save water*’. Charlotte said ‘*We all know that each and every day we need to drink a lot of water, maybe more than eight glasses of water, so … I drink four glasses of water because we need to save water*’. Similarly, Simpiwe (adolescent girl) said: ‘*To save water, I don’t waste water. For example, when I bath or brush my teeth, I don’t leave the taps open*’.

Co-researchers also anticipated that drought would recur. This resulted in proactive water-wise actions. For instance, Kutlo (adolescent girl) said: ‘*When we have lots of rain, water has become more useful. We need to save water*’. Angel (adolescent girl) said: ‘*I need to fill full the tanks so when there is a drought, I can get water from the tank*’. Similarly, KidEazy (adolescent boy) said: ‘*I have to be prepared. What I would do to be prepared, is to save water with cans, bottles, or maybe buy a tank. That will save me water for the future*.’

In addition, co-researchers emphasised that it was important to ‘*stay literate and more informed on how to handle the situation*’ (Zar, adolescent boy). This involved accessing information that could inform water conservation and acting on this information. For instance, Precious (adolescent girl) researched how to improve on what she was already doing to conserve water: ‘*Save water when bathing … when brushing your teeth, you open the tap [and] then you close it, you don’t keep it running. Then research, you do more research about drought to know and to have more information what drought is so that you can have ideas*’. Similarly, Zodwa (adolescent girl) said: *‘I have to be realistic, face the truth, understand that here is drought, find more information, research, find solutions … I can go to the internet, yes, there’s sometimes mention of the solutions, the things that you can do when there is drought*’.

#### 3.1.2. Collaborative Efforts to Conserve Water

Co-researchers were generally not alone in their efforts to conserve water: ‘*Some friends and family just like make a group and [we] talk about how we can save water*’ (Charlotte, adolescent girl). Co-conservation included family and friends acknowledging the reality of drought and shared valuing of water. Toni (adolescent boy) explained this as: ‘*To be OK when there’s a drought, it means that maybe at home they’ve saved water; I’ve got a little water to drink and that gives me energy to go to school’*. Others referred to how their families rationed water: ‘*We will use one bath water … my father is going to start first, then my mother, then my other brothers and sisters and then myself… We’re all going to use one water which is not right, but we have to do it because we don’t have a choice*’ (Junior, adolescent boy). Family members also taught one another to save water: ‘*My grandparents always taught us to save water... we have four drums [tanks] that has water … tanks make me feel OK because I just know that if I need water I can go to that tank*’ (Gwanele, adolescent girl) and ‘*At my house, we no longer leave the taps running. We won’t any longer allow my younger siblings to play in the water like they used to before. So, I think now we are more cautious*’ (Fission, adolescent boy).

There was isolated mention of the community collaboratively conserving water. In this regard, Lefa (adolescent boy) said: ‘*One thing that I’ve seen that drought does, it makes the community to come together. That is one advantage … we all came together... Not crying about the service delivery but of one thing that we all needed to survive in this world, which is water*.’ Co-researchers were of the opinion that they had a duty to support their community to conserve water: ‘*Be useful in your community … when you have a plan or solution to help overcome drought, tell everyone so that they can use it and work together*’ (Tshiamo, adolescent boy). Mostly adolescents supporting the community to conserve water entailed passing on information relating to drought and its management. Fission (adolescent boy) explained it like this: ‘*We see all these things [how to manage drought] on the TV and we have smartphones. We [re]search about these things and at school they teach us about these things, so yeah, that’s how we get information … some of the things I know, I learnt them from my parents. So, if I teach other older people that are doing the same things that my parents are doing, it doesn’t change… unless I add the new things I have learnt from the internet and from TV and from school and all those things, because, like our parents, some of our community are not educated*’.

In addition to passing on information, some believed that they needed to speak up when they saw water being wasted: ‘*I am thinking people must stop wasting water... If you see a child playing with a water, please stop him or her because water can be trouble in our area*’ (Gugu, adolescent girl). However, one co-researcher (Nocebo; adolescent girl), was sceptical. In her experience, the success of teaching peers and older community members to conserve water was temporary: ‘*We been trying but they don’t listen to us. Maybe they will do that thing for a week or so and then after two weeks they are not doing it anymore. Then we gave up, because they are not listening to us. They think we are just kids … we know nothing. So, that’s why we gave up*.’

Lastly, there was passing mention of government efforts to support households to conserve water by regulating the supply of water. KidEazy explained it as follows: ‘*We woke up in the morning; when you turn the tap on … there was no water … I think it was some sort of a strategy to save water … they [government] told us there was not enough water, so they were trying to save it … I think they were taking it during the day because they were thinking that everyone is at work … children are at school. So, there is no one who will need water that much. So, when the sun set, that’s when they would bring it back because people are back … they will want to bath, cook*.’ Similarly, there was passing mention that adolescents and their families supported these efforts. Precious said: ‘*Like the South African Department of Water … you have to respect their decision so that we can work together. Unite so we can have better solutions*.’

### 3.2. Health-Promoting Strategies

Health-promoting strategies were active efforts to buffer the negative health and wellbeing impacts associated with drought. As with water-conserving strategies, some of these efforts were adolescent-driven and some collaborative. Whilst the distal social ecology (i.e., government) endeavoured to partner in health promotion, adolescents considered their contributions less effective than those of the immediate social ecology (i.e., friends and family).

#### 3.2.1. Salutary Adolescent Actions

To sustain their health and wellbeing during drought-challenged times, co-researchers followed a healthy diet, exercised, and/or engaged in faith-based practices or expressive activity. They believed that these strategies would maintain their bodily and mental health despite the physical and psychosocial hardships that they associated with drought. Sithembiso (adolescent girl) said: ‘*I exercise… I eat healthy food each and every day because I want to keep my body healthy and strong. By eating healthy…and exercising during drought, I want to make sure that I don’t faint, even at school, when the temperature is so high and there is no water*’. Likewise, Njabulo (adolescent boy) said: ‘*Going to gym will help your body stay healthy … even though you don’t have enough water in your body*’. There was explicit mention of the protective mental health value of physical and spiritual activity. For instance, ‘*I exercise … jogging…some push-ups, sit-ups, you know, keeping myself busy, then I won’t think about drought and worry much*’ (Precious, adolescent girl); ‘*If I’m not feeling well about the drought, I just write*’ (Hakeem, adolescent boy); and ‘*I’m a Christian, so I pray. I pray to God that he helps me through drought and other things… and usually he does … [and] to play soccer or go to training it relaxes my mind … makes me forget about my problems … when I’m thinking too much, I listen to good music and then it relaxes my mind*’ (Junior, adolescent boy).

In some instances, drought conditions meant that co-researchers had to alter what they usually did to maintain their health. A case in point was the comment by Fission (adolescent boy): “*When there’s drought, I don’t play basketball like I usually do because I’m scared of being dehydrated. Instead of playing basketball, I just sit at home, read my comic books, watch movies, listen to music … Those are the things that I do to keep me strong emotionally*”. ‘Immaculate (adolescent girl) also had to alter her health-promoting actions: ‘*When there’s a drought … I usually read books, go to church … that helps me a lot because it improves my way of thinking … [and] I get to forget about what is happening … I need to eat a lot of fruit and vegetables … and they are not there when there’s a drought, so I usually go to the sports ground to keep—just to keep my body healthy*’. These examples point to salutary actions sometimes requiring improvisation and flexibility.

As can be seen from the excerpts, the salutary actions did not appear to be gender specific. Adolescents confirmed this. For instance, Najabulo (adolescent boy) said: ‘*What helps me will also help girls. There’s nothing different.*’ Similarly, Precious (adolescent girl) commented: ‘*It [the enabling strategies she had mentioned] helps everyone, because what I’ve put here it doesn’t discriminate: exercise [is] for everyone; friends, everyone; alerts for everyone*.’

#### 3.2.2. The Immediate and Distal Social Ecology Co-Facilitate Adolescent Health

Friends and family co-supported co-researchers’ efforts to maintain their health and wellbeing in the face of drought. Charlotte said: ‘*We [friends] … chill out, talk about other things, [like] good memories … [we] don’t just focus on how this drought is affecting us a lot … that is how we support each other*’. Similarly, Fission (adolescent boy) said: ‘*My family is very supportive … they give you that hope … (by) making sure we are still connected to God, our ancestors … they tell us that, uh, our ancestors will always look after us. So that somehow gives us strength and gives us hope’*.

Although co-researchers typically referred to support during the time of drought, there was mention of health-promoting advice that predated the experience of drought. Co-researchers’ recall of such historic support informed how they protected their health in the face of drought. For instance, Thato (adolescent girl) reported: ‘*My first rule in my mind is do not think negative… my father taught me this when I was young*’. KidEasy (adolescent boy) also emphasised the importance of being hopeful. Like Thato, this had been passed on to him by a parent: ‘*You see, I believe in my mother. Everything she tells me like I believe will happen. So, she used to tell me that everything was going to be OK*.’

In addition to gaining support from people in their immediate social ecology, co-researchers experienced that contributing to their family and community had personal health-promoting benefits. In this regard, Thembi (adolescent girl) said: ‘*It’s helping me by helping others who suffer from drought. And also [by helping] young children… to keep their minds positive*’. Similarly, Bonele (adolescent girl) said: ‘*I also play with young kids, for fun, and that’s what also relieves me … I help old people when they go and collect water in the river or some other location that is near and has water*.’ According to Toni (adolescent boy), the benefits of helping others was not limited to the present: ‘*Love your community … and respect everyone … maybe one day that person will help you*’.

Lastly, there was some mention of action taken by government to supply the adolescents and their community with water. However, co-researchers were ambivalent about the health-promoting value of these strategies. For instance, Kutlo reported: ‘*They [government] simply take trucks and then simply take [water] to this place [her community] … at the same time you don’t feel comfortable about this water as already … wondering where did they get the water from, is this truck clean?*’ In addition, inadequate water provisions were associated with community dissent: ‘*The trucks that were supplying us with water, like they didn’t have enough water. So, people ended up fighting for water because everyone wanted water*’ (Fission, adolescent boy).

## 4. Discussion

This article is concerned with the personal and social-ecological factors that protect the health and wellbeing of a group of South African adolescents exposed to drought, and the contextual responsiveness of these resilience-enabling factors. That focus is overdue, given the historic inattention to the health and wellbeing of African youth who are exposed to natural disasters in general and to drought in specific [56]. As communicated by the adolescent co-researchers, youth resilience to drought is grounded in proactive and reactive strategies—all constructive—that enable water conservation and protect health (physical and mental). In a water-scarce country such as South Africa where drought is likely to be a regular phenomenon [10], proactive strategies are preferable to reactive ones and should be purposively encouraged. One way to do so would be to encourage multidisciplinary programs to store water. The active participation of young Africans will be crucial to the success of such programs, as will national and global support.

As presaged by social-ecological theories of resilience [15,18], pre-existing South African child and youth resilience studies [20], and the handful of studies of Australian youth resilience to drought [4,6,7,31], the enabling strategies reported by adolescent co-researchers were championed by young people and their social ecologies. This overlap suggests that interventions toward enabling adolescent resilience to drought, also in South Africa, must support a social ecology to accept co-responsibility for adolescent resilience to drought. Doing so is not dismissive of the value of adolescent agency, as was experienced by the adolescent who reported community disregard for youth efforts to support collective resilience. Rather, it is about discouraging expectations that youth manage drought-related challenges on their own, or that adults prescribe youth responses. Adolescent health and wellbeing will benefit from families, schools, faith-based organisations and other community structures, and local and other government working with young people to sustain youth-identified factors that buffer drought-related challenges and inviting youth guidance on additional protective factors that should be enabled.

Despite the aforementioned broad commonality between our findings and pre-existing studies of youth resilience to drought, there were contextually sensitive facets to the reported strategies. These contextually aligned nuances are a reminder of the importance of theorising youth resilience—also to drought—in socioculturally relevant ways [18,19]. For instance, the youths’ sense of responsibility to educate their social ecology on how best to manage drought-related risks likely reflects the low rates of high school completion among adults in their community (i.e., only 31% [44]). These low rates probably relate to South Africa’s Apartheid history and the socially engineered obstruction of African people’s access to education [57], and African adults’ subsequent appeals to youth to valorise education and to use their education to uplift families and communities [20]. Similarly, youths’ references to the personal and collective benefits of helping others fit well with traditional African values of interdependence and resource-sharing [51]. These references could, however, also point to South African realities of structural violence [58], and associated experiences of unavailable, inaccessible, or inadequate services and other institutional/government supports [59,60]. In the face of social and structural inequality, it is possible that supportive interdependence and other-mindedness—also to the challenges of drought—are a protective response to collective experiences of marginalisation and oppression and recognition of the need to protect oneself and those whom one is connected to.

Further, unlike the Australian youth in the Carnie et al. study [31], there was no reference to formal or structural resources that traditionally support or respond to health-seeking (e.g., support groups, mental health services). Similarly, unlike the study with drought-challenged youth from Botswana [30], there was no reference to government responsiveness to heightened food insecurity during drought (e.g., government-funded feeding schemes). Instead, reference to structural resources was scant. When there was reference to structural resources or government intervention, it was typically sceptical. This fits with lived experiences of structural disadvantage. Although the limited mention of structural resources could perhaps relate to adolescents being more focused on resources such as families or friends that are prominent in their everyday lives [20], it is likely that structural resources were largely unavailable in this resource-constrained community [59,60]. Given this, social justice initiatives and concomitant redress of resource inequity will be essential to youth resilience to drought in resource-constrained communities, just as such initiatives are essential to the resilience of youth in non-drought-challenged but structurally disadvantaged communities [61].

If the relative silence about structural resources was related to their absence (rather than their invisibility to youth [20]), then it is striking that youth made no reference to personal or collaborative efforts to gain structural support. There was not even any mention of youth asking for help from school-based staff, with whom they must have had regular contact. Whilst it is possible that the absence of efforts to gain structural or formal support could fit with a preference for self-reliance [2], it is also possible that youth were unsure how to seek help from formal or institutional sources. To this end, interventions to advance youth resilience to drought should encourage help-seeking, not only by youth but also by their communities. Supporting adolescents and their communities to map formal or institutional resources and research how best to access these resources might be a useful starting point. Again, given the likelihood that drought will recur, proactive help-seeking is particularly important.

In summary, the current article is the first to document youth resilience to drought in an African context, as explained by adolescents themselves. Its contribution lies in the youth-directed finding that *both* personal and social-ecological resources matter for the resilience of youth exposed to drought. The resilience literature has generally marginalised the voices, so to speak, of youth [13], more especially youth living in low- and middle-income countries in the Southern hemisphere [14]. These African youth voices point to resilience-enabling solutions for drought-related challenges that are part practical (i.e., water-focused), part biopsychosocial (i.e., supportive of health and wellbeing), and reliant on youth and their social-ecologies collaborating. Whilst previous studies of youth resilience to drought [4,6,7,30,31], and the original analyses of the data from *Patterns of resilience among young people in a community affected by drought* study [35,36,37,38,39], identified personal and social-ecological resources, they neglected to explicate that personal and social-ecological resources co-facilitate youth resilience to drought. They also overlooked how situational and cultural context shape the resources that youth identify as protective of health and wellbeing in times of drought. Inattention to these complexities will undermine resilience-enabling solutions for drought-related challenges. In contrast, understanding that youth resilience to drought is a multifaceted, dynamic, co-facilitated process should encourage collaboration across disciplines and sectors that enables health, wellbeing and water saving in contextually responsive ways.

### Limitations

As noted elsewhere [15,19], resilience accounts are limited if they do not draw on longitudinal data. Further, at the time of the study, recent rainfall had brought some relief to the water shortages in the community. Had we worked longitudinally with this group and elicited their insights when drought conditions were more severe (as has happened again since the 2017 work), it is possible that their accounts of what protects adolescents from drought-related risks might have been somewhat different. Additionally, although all the co-researchers were willing to communicate with us in English, it is probable that communicating in their mother-tongue would have yielded even richer insights. The fact that adolescent boys and girls believed that the enabling strategies were gender neutral, and that the same strategies were reported by adolescent boys and girls, was interesting. This is at odds with pre-existing findings that resilience processes are gendered [25]. One wonders whether this finding would have been different if the group had included adolescent mothers, or other adolescents who had been expected to endorse gendered identities or roles. Similarly, a study with a larger sample that was characterised by equal numbers of adolescent boys and girls from diverse social and physical ecologies might have resulted in insights different from the ones we report. Finally, given that qualitive findings invariably reflect participants’ subjective experiences, it would be valuable to complement the current study with a follow-up one that surveys a larger sample of drought-affected youth to investigate the extent to which their resilience to drought reflects enabling actions that draw on individual and collective input, and how these vary across diverse situational and cultural contexts. The results will be useful to the development of responsive resilience-to-drought interventions.

## 5. Conclusions

Despite the above-mentioned limitations, our study offers insight into what enables resilience for a group of adolescents with first-hand experience of drought-related challenges. The passing on of their insights advances much needed adolescent-directed accounts of resilience [13,14]. Moreover, with predictions that water insecurity is rising globally [3], their insights give direction to those wanting to champion youth resilience to drought. They draw attention to the salience of personal and social-ecological resources and how contextual influences nuance the expression and availability of these resources. Essentially, the findings caution that anything less than systemic, contextually responsive, socially just interventions will be a disservice to youth challenged by drought.
